# Sonographic Extracranial–Intracranial Bypass Monitoring: Technical Evolution and Current Evidence

**DOI:** 10.3390/brainsci16070672

**Published:** 2026-06-26

**Authors:** Eric A. Grin, Erez Nossek

**Affiliations:** Department of Neurosurgery, NYU Grossman School of Medicine, New York, NY 10016, USA

**Keywords:** cerebral revascularization, cerebrovascular disorders, ultrasonography, transcranial Doppler, intracranial aneurysm, extracranial–intracranial bypass, cerebral ischemia, cranioplasty

## Abstract

**Highlights:**

**What are the main findings?**

- Sonographic imaging of EC-IC bypass has evolved from indirect extracranial Doppler and intraoperative microvascular techniques to trans-sonolucent cranioplasty ultrasonography (TCUS), which enables direct, radiation-free visualization of the bypass anastomosis through a permanently implanted acoustic window.
- TCUS thus far has demonstrated complete concordance with formal angiography for bypass patency assessment in longitudinal series and can detect qualitative hemodynamic changes, including waveform attenuation and vessel caliber reduction, that correspond to angiographically confirmed anatomical findings.

**What are the implications of the main findings?**

- TCUS offers a safe, noninvasive, and potentially cost-effective alternative to serial DSA, CTA, or MRA for long-term bypass surveillance, with particular value in patients requiring repeated monitoring over many years, including those treated for complex aneurysms with parent vessel sacrifice.
- Transition to quantitative TCUS protocols, standardization of acquisition and training, and prospective validation in high-flow and aneurysm-specific bypass populations represent the critical next steps toward establishing TCUS as a potential primary modality for routine postoperative graft surveillance.

**Abstract:**

Extracranial–intracranial (EC-IC) bypass surgery remains an essential tool in the management of complex cerebrovascular disease, including chronic-ischemic hypoperfused territory as well as intracranial aneurysms requiring parent vessel sacrifice. Reliable postoperative surveillance of graft patency and hemodynamic function is central to long-term success. Sonographic imaging has evolved over four decades from indirect extracranial Doppler waveform analysis through intraoperative microvascular Doppler, quantitative duplex ultrasonography, and transcranial Doppler assessment of intracranial hemodynamics to the contemporary development of trans-sonolucent cranioplasty ultrasonography (TCUS). TCUS, enabled by the replacement of the autologous bone flap with an acoustically transparent polymethyl methacrylate (PMMA) cranioplasty, permits direct, real-time visualization of the anastomosis and surrounding intracranial vasculature. This can be performed at the bedside and in the outpatient setting, without radiation or contrast. Current evidence, drawn largely from small series and early multicenter experience, positions TCUS as a reliable and potentially cost-effective complement to digital subtraction angiography for serial bypass surveillance, though prospective validation remains needed. This review traces the full arc of sonographic bypass monitoring and addresses current evidence, technical considerations, established quantitative parameters, and directions for future investigation.

## 1. Introduction

Surgical revascularization through extracranial–intracranial bypass has been performed for more than half a century, and although its indications have evolved considerably, it remains one of the most technically demanding procedures in cerebrovascular neurosurgery [1]. Direct anastomosis of the superficial temporal artery (STA) to the middle cerebral artery (MCA) constitutes the most commonly performed flow augmentation bypass and has demonstrated clear clinical benefit in moyamoya disease [2,3]. Flow replacement bypass with interposition grafts using the radial artery or saphenous vein are required when flow demands exceed the STA’s capacity, most notably in the management of complex intracranial aneurysms that cannot be directly clipped or coiled and require sacrifice of the parent vessel [4,5,6]. In both contexts, the technical success of the anastomosis and its sustained patency over time are the primary determinants of clinical outcome.

Monitoring graft patency is accordingly a central component of postoperative care. Bypass failure, whether from early thrombosis due to a technical error, progressive stenosis, or late occlusion, can result in ischemic stroke. In patients whose bypass was constructed to replace a sacrificed vessel, graft failure may be catastrophic [3,7]. Traditional surveillance has depended on catheter-based digital subtraction angiography (DSA), which provides unmatched spatial resolution and hemodynamic detail but is invasive, costly, radiation-generating, and carries procedural risks that limit its suitability for repeated long-term use [8,9]. Computed tomography angiography (CTA) is less invasive but still involves ionizing radiation and iodinated contrast, restricting repeated application, particularly in younger patients and in those with renal impairment [8,9,10]. Magnetic resonance angiography (MRA) avoids radiation but involves lengthy acquisition times and, for smaller anastomoses, spatial resolution that may be insufficient for confident patency assessment [11].

Ultrasonography has long been recognized as an appealing alternative for bypass surveillance, offering noninvasive real-time assessment without radiation, contrast, or the need for patient transport [12]. From the earliest extracranial Doppler examinations of the 1980s through modern quantitative duplex imaging, ultrasound has contributed meaningfully to both intraoperative decision-making and postoperative monitoring [13,14]. Its fundamental limitation is the skull’s high acoustic impedance, which limits ultrasound transmission to the intracranial compartment once the bone flap is replaced. Historically, this has confined postoperative ultrasound assessment either to extracranial vessels or to the brief window of an open craniotomy [15,16]. The development of sonolucent cranioplasty materials over the past decade has been transformative: elective replacement of the bone flap with a sonolucent PMMA prosthesis can create a permanent acoustic window enabling direct intracranial visualization indefinitely after surgery [16,17,18].

This narrative review provides a comprehensive account of the evolution and current evidence for sonographic imaging in EC-IC bypass surgery. Studies were selected at the authors’ discretion to represent the historical development and current state of the field; no formal systematic-review methodology (e.g., PRISMA) or quantitative meta-analysis was applied, and formal risk-of-bias assessment was not undertaken. This review proceeds chronologically from the earliest extracranial Doppler and intraoperative microvascular approaches through contemporary quantitative duplex surveillance, culminating in a detailed treatment of trans-sonolucent cranioplasty ultrasonography (TCUS), which represents an important emerging advance in this domain. Technical considerations, established quantitative parameters, comparisons with alternative imaging modalities, and future directions are addressed throughout.

## 2. Historical Foundations: Extracranial Doppler and the Transcranial Revolution

### 2.1. Early Extracranial Doppler Assessment

The systematic application of ultrasound to EC-IC bypass monitoring began in the early 1980s [19,20]. In 1983, Mehdorn et al. published one of the first reports demonstrating that Doppler sonography could reliably determine patency and functional status of EC-IC anastomoses [19]. Their approach was indirect: continuous-wave Doppler applied over the pre-auricular STA assessed flow velocity and waveform morphology before and after surgery. A functioning bypass was identified by two cardinal findings: (1) an increase in STA peak velocity relative to preoperative baseline, and (2) a transformation of the STA flow waveform from a high-resistance external carotid pattern (with minimal diastolic flow) to a low-resistance internal carotid pattern (with sustained diastolic flow), reflecting the graft’s new connection to the low-resistance intracranial circulation [19]. Combined with dynamic CT for qualitative perfusion assessment, Mehdorn argued that this approach could render conventional angiography unnecessary in routine cases [19].

While useful, this technique also carried fundamental constraints. Since there was no direct visualization of the anastomotic site or of intracranial vessels, all conclusions about bypass function were inferential, derived from hemodynamic changes in the extracranial donor vessel. This was a purely spectral method. Operators worked from audio signals and velocity tracings without any anatomical imaging. Critically, extracranial Doppler could not distinguish between flow in the bypass limb and flow in non-anastomosed STA branches, a limitation that has implications for false-positive patency assessment that remain relevant even in contemporary practice, as discussed below. There was also no capacity to assess the intracranial circulation directly, nor to quantify volumetric flow [19].

### 2.2. Transcranial Doppler Sonography

Rune Aaslid’s introduction of pulsed-wave transcranial Doppler (TCD) in 1982 was a major breakthrough in non-invasive intracranial hemodynamic assessment [21]. A 2 MHz probe transmitted through thin areas of the temporal bone enabled, for the first time, direct insonation of intracranial vessels without craniotomy. Harders and Gilsbach applied this technology specifically to bypass surgery in their seminal 1984–1985 publications, providing the first systematic characterization of pre- and postoperative intracranial hemodynamics in bypass patients [22,23]. Their critical methodological innovation was the STA compression test: by temporarily occluding the extracranial donor STA and observing resultant changes in MCA flow velocities measured by TCD, they could demonstrate the functional contribution of the bypass to intracranial perfusion [22]. A graft that was patent but hemodynamically inconsequential (i.e., supplying little flow to the brain) would produce negligible velocity change in the MCA upon compression; a functionally important bypass would produce a measurable flow reduction. This permitted assessment of bypass efficacy, not merely patency, without formal angiography [22,24].

Even so, in this era, TCD was non-imaging (niTCD) and entirely operator-dependent. Vessel identification relied on standardized depth measurements from the skull surface, characteristic waveform recognition, and compression maneuvers rather than any direct anatomical visualization [22]. Approximately 8–20% of patients, with rates highest in elderly women and in patients of East Asian or African ancestry, had inadequate temporal bone acoustic windows precluding examination altogether, with no available method of compensation [25,26]. Additionally, despite its ability to detect flow velocity changes downstream of the anastomosis, TCD could not directly visualize the anastomotic site itself. Assessment thus remained indirect: the operator inferred anastomotic function from intracranial hemodynamic effects rather than by observing the bypass directly [27].

Nonetheless, TCD established an enduring methodological principle in bypass monitoring: the combination of extracranial donor vessel assessment with intracranial flow velocity measurement could provide complementary hemodynamic information, with neither technique alone being sufficient. Contemporary transcranial color-coded duplex sonography (TCCS) builds on this foundation by adding B-mode imaging and color-flow mapping to spectral velocity data, enabling classification of intracranial hemodynamic patterns—for instance, distinguishing cases in which the entire MCA territory is dependent on bypass flow from cases in which native collateral circulation remains contributory [27,28,29].

## 3. Intraoperative Microvascular Doppler Sonography

### 3.1. Development and Core Capabilities

Parallel to the development of transcranial approaches, intraoperative microvascular Doppler technology with miniaturized probes emerged as a critical surgical adjunct in the 1980s. In 1984, Gilsbach et al. demonstrated that high-frequency (20 MHz) pulsed-wave Doppler with 1 mm diameter probes placed directly on exposed vessels could reliably assess vessels as small as 1 mm in diameter, precisely the caliber of typical bypass anastomoses [22,23]. A critical clinical observation was that nearly 10% of anastomoses that appeared technically successful to direct visual inspection proved hemodynamically unsatisfactory on Doppler examination, allowing immediate intraoperative correction before closure [22,23].

The allowance of immediate real-time detection of anastomotic problems invisible to the naked eye, including stenosis from suture tension, occlusion from thrombosis, or kinking of the donor vessel, was a tremendous contribution to the field of cerebrovascular neurosurgery [22,23]. Vessel patency, flow direction (distinguishing antegrade from retrograde flow), resistance changes, and the presence of high-frequency turbulent signals indicating stenosis could all be assessed [30]. By the early 1990s, tandem probe configurations permitted simultaneous comparison of pre- and post-anastomotic waveforms, allowing detection of anastomotic narrowing as small as 5% [22,30,31].

The technique proved particularly valuable in the setting of complex intracranial aneurysms. Bailes’ landmark 1997 study demonstrated that 31% of patients undergoing aneurysm procedures had parent artery or branch artery stenosis or occlusion detected by intraoperative Doppler that prompted immediate clip repositioning [30,31]. Subsequent studies confirmed the high detection rate of problems invisible to visual inspection, a rate that was particularly high for middle cerebral artery aneurysms (38.6%) and in patients with subarachnoid hemorrhage (36%) [30,32]. A 20 MHz probe could reliably insonate all major vessels of the circle of Willis, their principal branches, and even perforating arteries like lenticulostriate and thalamoperforating vessels with diameters below 1 mm [31]. In aneurysm surgery, where perforator compromise can produce profound neurological deficits that only become apparent postoperatively, the ability to detect and correct such problems in real time, before closure, was of considerable clinical consequence [30].

For bypasses performed in the context of aneurysm management, in addition to assessing patency, intraoperative Doppler guided the timing of parent vessel sacrifice. In 2000, Badie et al. described the use of intraoperative duplex sonography during EC-IC bypass for fusiform posterior circulation aneurysms, demonstrating that graft patency and flow direction could be rapidly confirmed at the bedside of the operative field, providing the necessary information for deciding when to occlude the parent vessel without requiring formal intraoperative DSA with its associated time cost, radiation exposure, and logistical complexity [33]. This capacity for real-time hemodynamic decision-making remains one of ultrasonography’s most clinically impactful contributions.

### 3.2. The Transit-Time Ultrasonic Flow Probe

A transformative advance in intraoperative hemodynamic assessment came with the clinical application of the transit-time ultrasonic flow probe, extensively developed and validated by Charbel and colleagues at the University of Illinois at Chicago [34]. Unlike spectral Doppler, which measures flow velocity, the transit-time probe is placed circumferentially around the vessel of interest and directly measures volumetric blood flow in milliliters per minute, providing both pulsatile flow curves and mean flow values in real time without requiring vessel diameter assumptions or angle correction [34,35]. This technology enabled a fundamentally new paradigm in bypass surgery: flow replacement bypass, in which the graft is not simply confirmed as patent but is verified to deliver flow matched to the measured hemodynamic deficit.

Amin-Hanjani et al. demonstrated that baseline intraoperative flow measurements in the vessel to be sacrificed could predict the flow required for adequate replacement. For terminal aneurysms, target flows varied by territory: the MCA required approximately 50 mL/min, the PICA 13 mL/min, the PCA 33 mL/min, and the SCA 10 mL/min [36]. For proximal ICA aneurysms, the flow deficit during temporary occlusion averaged 26 mL/min, representing a 44% reduction from baseline [36].

Post-anastomotic probe measurements confirmed not only patency but adequate flow delivery in all cases, enabling rational selection among donor options (STA, occipital artery, or interposition vein graft) based on objective hemodynamic requirements rather than anatomical assumptions alone [37]. Large outcome series have validated this flow-guided approach: in the Nussbaum series of 126 patients treated over nearly two decades, low-flow bypass achieved 98.8% patency at 12 months with 90.4% good functional outcomes, while the Sekhar series of 233 bypasses reported 92% long-term patency and 81% good outcomes at three months [4,38]. The cut flow index (CFI), defined as the ratio of post-anastomotic to pre-anastomotic cut flow measurements, emerged from this work as a standardized intraoperative metric with predictive value for long-term patency, with a threshold of 0.5 associated with favorable outcomes in large series [37,39].

### 3.3. Limitations of the Intraoperative Era

Despite its advantages, intraoperative microvascular Doppler had fundamental constraints. Again, it was purely spectral, lacking B-mode imaging, anatomical visualization, or the capacity to see the vessel wall, the anastomotic suture line, or intraluminal thrombus [40]. The correlation between measured velocity and actual volumetric flow was imperfect, with reported errors of 10–18% when Doppler velocity was compared with electromagnetic flowmeter measurements [40,41,42]. Quantitative volumetric flow measurement was not possible without vessel diameter data that required imaging.

Most importantly, all monitoring was confined to the intraoperative period. Once the bone flap was replaced, the skull reconstituted its acoustic opacity and the anastomosis became largely hidden to ultrasound [43]. Long-term postoperative surveillance, which is needed to detect late thrombosis, assess graft maturation, or identify progressive stenosis over months to years, could not be accomplished by any ultrasound technique until the advent of sonolucent cranioplasty [17,44]. In the intervening decades, postoperative monitoring reverted to conventional angiographic imaging.

## 4. Postoperative Duplex Ultrasonography: Quantitative Assessment of Bypass Function

### 4.1. Extracranial Duplex Surveillance and Flow Parameters

The development of duplex ultrasonography, combining B-mode imaging with spectral Doppler and color-flow mapping, substantially advanced the postoperative assessment of EC-IC bypass grafts [45]. By measuring vessel diameter alongside flow velocity, volumetric flow rates could be calculated, enabling quantitative characterization of bypass function rather than simple patency determination [14,45]. This was particularly important for high-flow bypasses, where flow adequacy, not merely patency, determines whether the revascularization is hemodynamically sufficient.

For high-flow EC-IC bypasses using radial artery or saphenous vein interposition grafts, as are commonly required for complex intracranial aneurysms necessitating major vessel sacrifice, Morton et al. established normative flow parameters based on a large single-center experience of 80 grafts monitored over 8 years. Average flow volumes were approximately 133 mL/min for radial artery grafts and 160 mL/min for saphenous vein grafts, a difference that was not statistically significant [45]. Multiple variables were found to influence graft flow, including donor and recipient vessel selection, preoperative graft diameter, and postoperative hematocrit [46,47]. This work also identified a one-week average flow exceeding 200 mL/min as demonstrating 100% sensitivity for cerebral hyperperfusion syndrome, providing a quantitative ultrasound target for early identification and management of this serious complication [45].

For low-flow STA-MCA bypasses, Wang et al. characterized the temporal evolution of graft flow parameters in 131 operated hemispheres, measuring STA flow extracranially using quantitative duplex ultrasonography applied to the graft at its pre-cranial location, with assessments at preoperative baseline and at postoperative days 1 and 7, and at 3 and 6 months [13]. Mean flow in the STA graft increased dramatically from approximately 24 mL/min preoperatively to over 100 mL/min by postoperative day 7, followed by gradual stabilization over subsequent months. These flow values correlated strongly with bypass function as assessed angiographically by the Matsushima classification: grade A bypasses (reflecting excellent territory revascularization) averaged approximately 168 mL/min, grade B averaged 91 mL/min, and grade C averaged 42 mL/min. Optimal flow thresholds of 124.5 mL/min and 65.5 mL/min were identified as discriminating boundaries between Matsushima grades, with excellent agreement between duplex ultrasonography and DSA [13]. This work demonstrated that extracranial STA duplex could not only confirm patency but could also reliably predict the quality of bypass revascularization.

Morphological parameters also carry prognostic significance. Kim et al. showed that the ratio of postoperative to preoperative mean flow rate and cross-sectional diameter at one month, with cutoffs of 1.475 for mean flow rate and 1.15 for cross-sectional diameter, were the most reliable predictors of DSA-confirmed patency [48]. Nakamizo et al. established that an STA diameter of at least 1.8 mm at one year after surgery predicted improved cerebrovascular reserve with 96.6% positive predictive value, directly linking an ultrasonographic measurement to the ultimate physiological goal of hemodynamic augmentation [49].

### 4.2. Transcranial Doppler Velocity Criteria

TCD assessment of the intracranial MCA territory complements extracranial STA duplex by characterizing downstream hemodynamic effects of bypass function. For STA-MCA bypasses, Chen et al. established specific velocity-based patency thresholds through receiver-operating-characteristic analysis: peak systolic velocity (PSV) in the donor STA exceeding 49.0 cm/s, a postoperative-to-preoperative PSV ratio exceeding 1.218, and an operated-to-contralateral PSV ratio exceeding 1.082 each demonstrated good sensitivity and specificity for patent bypass [12]. In patent grafts, PSV increased significantly while pulsatility index decreased significantly compared to non-patent grafts, reflecting the transition from high-resistance to low-resistance flow that had been qualitatively described in the earliest extracranial Doppler studies of the 1980s [12,19,24,50].

Transcranial color-coded duplex sonography has extended this framework by enabling classification of intracranial hemodynamic patterns. Umemura et al. described three patterns: complete MCA territory dependence on bypass flow, partial dependence with independent M1 supply, and inefficient bypass contribution with collateral dominance [51]. Preoperative absence of collateral flow through the anterior communicating artery and impaired cerebrovascular reactivity predict hemodynamically useful postoperative bypass flow, providing a basis for using preoperative TCD findings in operative planning [52,53].

### 4.3. Detection of Complications

Beyond confirming patency and quantifying flow, duplex ultrasonography has demonstrated utility in the detection of specific postoperative complications. Graft occlusion is identified by absence of color Doppler signal and flat spectral waveforms [14,33]. Hyperperfusion syndrome, occurring in approximately 1–3% of EC-IC bypass cases, is heralded by sustained flow exceeding 200 mL/min over the first postoperative week, as established by Morton’s normative data, enabling preemptive blood pressure management and other prophylactic measures [45,46,47]. Graft stenosis can be identified by focally elevated velocities, turbulent flow signals, or waveform dampening distal to the site of narrowing [14,54,55].

For high-flow bypasses in aneurysm cases, saphenous vein grafts have been found to have higher occlusion rates than radial artery grafts in some series, emphasizing the importance of graft-type-specific surveillance protocols [3,56]. The dynamic nature of bypass hemodynamics in the early postoperative period, with the first postoperative week representing a particularly critical window, means that a single postoperative assessment is insufficient. Serial surveillance is necessary to capture evolving graft maturation and to identify delayed hemodynamic changes [3,14,57].

## 5. Trans-Sonolucent Cranioplasty Ultrasonography

### 5.1. Rationale and Background

**Figure 1:**
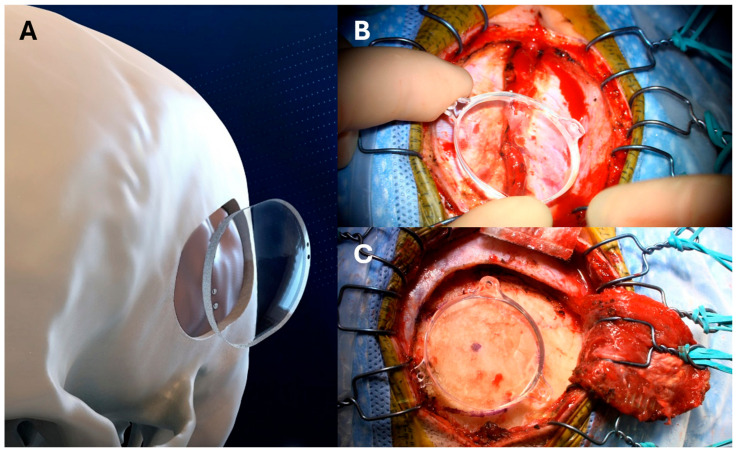
(**A**) Rendered model demonstrating the positioning of the sonolucent cranioplasty implant over the craniotomy site. (**B**) Intraoperative visualization of the implant held over the surgical field following STA dissection; the donor vessel is visible beneath the disc. (**C**) Intraoperative assessment of implant sizing on the exposed calvarium prior to craniotomy. The fiducial marker (purple dot) indicates the planned site for the microvascular anastomosis, which was localized via stereotactic neuronavigation. Images are original and obtained from patients at the authors’ institution with appropriate informed consent.

The fundamental limitation of all postoperative ultrasound bypass assessment prior to the current era was the skull’s acoustic impermeability once the bone flap was replaced. Extracranial duplex could assess only the donor vessel and its hemodynamic transformation; TCD could assess intracranial velocities through the temporal bone in patients with adequate windows but could not directly visualize the anastomosis; and none of these techniques could provide direct anatomical imaging of the bypass at its most critical site—the anastomosis itself [14]. TCUS resolves this constraint by substituting an acoustically transparent prosthetic implant for the autologous bone flap, creating a permanent, anatomically targeted acoustic window (Figure 1) [17,58].

The conceptual and material science foundation was established by studies in 2019 demonstrating that polymethyl methacrylate (PMMA) cranioplasty implants produced ultrasound images with spatial fidelity comparable to CT and MRI, with negligible beam degradation or distortion across all imaging planes [59]. Cadaveric work confirmed that both clear and opaque PMMA, as well as polyetheretherketone (PEEK), were sonolucent, while autologous bone and porous polyethylene were not [44]. The first clinical application was reported in 2020 by Hadley et al., who described elective implantation of a sonolucent PMMA cranioplasty in a moyamoya patient undergoing STA-MCA bypass, enabling bedside confirmation of bypass patency postoperatively [17]. In the same year, Flores et al. reported safety, feasibility, and patient-reported outcomes in a small series and estimated TCUS follow-up imaging costs at approximately $900 per evaluation, compared to $11,000 for conventional imaging modalities, a cost differential that, while based on estimated rather than formally validated cost-effectiveness data, suggests potential economic advantages for programs performing high volumes of bypass surgery [58,60].

### 5.2. Technical Aspects

The sonolucent PMMA implants currently in widest clinical use, most notably the ClearFit disc (Longeviti Neuro Solutions), are available in standardized dimensions: a 3 cm round implant for single-recipient anastomoses and a 4 × 5 cm disc for larger craniotomies with multiple potential recipient vessels [16,18]. Fixation techniques have evolved from early integral fixation wings to the current use of titanium plates and screws. When the proximity of the anastomosis to the overlying implant raises concern for graft compression, the inner surface can be thinned by drilling intraoperatively to create adequate clearance [16,61].

**Figure 2:**
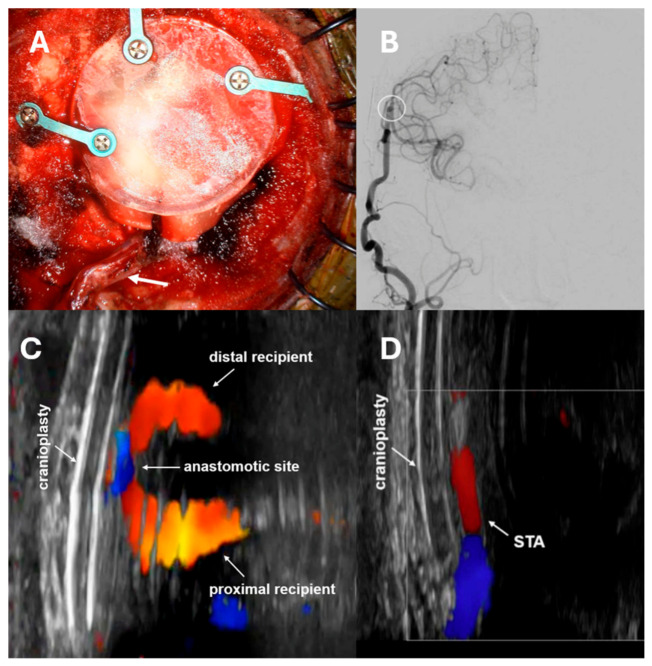
TCUS performed for an STA-MCA bypass. (**A**) The sonolucent cranioplasty is fixed in place with titanium plates and screws, and the inferior burr hole on the skull was enlarged to create space for the STA (arrow) to cross intracranially. (**B**) Formal DSA 2 years postoperatively demonstrates a large donor STA and patent anastomosis (circle) with robust distal flow into the MCA and ACA territories. Outpatient TCUS over 1 year postoperatively demonstrating (**C**) focal color aliasing at the anastomotic site, with distal and proximal flow within the MCA branch with strong color signal, and (**D**) flow in the STA passing beneath the sonolucent cranioplasty. Images are original and were obtained from patients at the authors’ institution with appropriate informed consent.

TCUS examinations can be performed using handheld or conventional ultrasound devices with a linear transducer placed directly over the implant [58,62]. Initial scanning establishes visualization of key anatomical landmarks: the implant itself, the extracranial donor STA passing beneath it, the anastomotic site, and the MCA proximal and distal to the anastomosis (Figure 2) [17]. Color Doppler permits direct real-time visualization of flow through the anastomosis, while spectral Doppler with angle correction enables waveform analysis and quantitative velocity measurement [48]. Robust patency is characterized by strong, continuous, well-defined arterial waveforms in the STA with preserved diastolic flow and clear color signal through the anastomosis and into the recipient MCA territory [16,18,48,62].

Several practical and temporal constraints govern image acquisition. Because ultrasound does not penetrate air or fluid collections, the implant manufacturer recommends that the first postoperative ultrasound be deferred until at least 24 to 48 h after surgery in order to allow resorption of subgaleal and intracranial air and epithelialization of the surgical wound. TCUS is therefore generally not suitable for immediate intraoperative-to-early-postoperative monitoring in the first 24 h. Topical hemostatic agents, collagen-based overlays, and certain dural substitutes may further interfere with ultrasound penetration, and the fresh incision itself can be difficult to scan across, sometimes requiring additional gel as a standoff.

Since ultrasound does not transmit through bone, accurate probe alignment over the implant footprint is essential; acoustic shadowing at the implant margins can be used to confirm probe position. A low-frequency linear transducer (e.g., 9 MHz) with a “carotid” preset is often best suited to the shallow (1–3 cm) depth of EC-IC bypass imaging, whereas a sector (phased-array) probe is preferable for deeper neurovascular structures. Point-of-care and handheld systems can provide immediate bedside results when symptoms change or for routine surveillance, while higher-resolution radiology-console systems may be reserved for situations requiring superior image quality.

The primary technical challenge is prosthesis curvature, which can impair optimal transducer-scalp contact and image acquisition quality [63]. Additional sources of artifact include early postoperative pneumocephalus, metallic plating systems, and dural sealants [58,62]. Despite these limitations, measurements of intracranial structures through PMMA cranioplasty have demonstrated excellent correlation with CT imaging in validation studies, and images acquired through the implant show spatial relationships superimposable to those obtained through direct dural contact [59,63].

**Figure 3:**
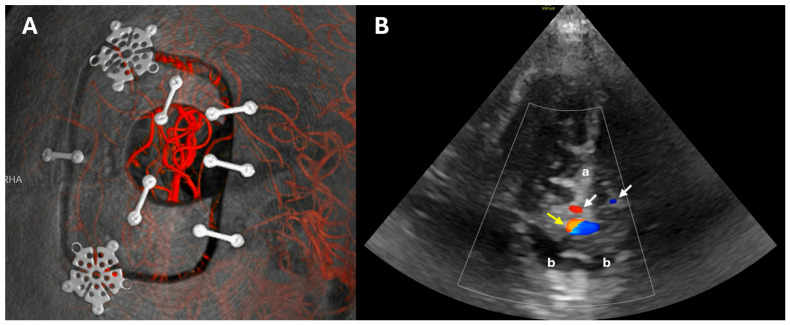
TCUS to assess ACA-ACA (A3-A3) side-to-side bypass for a complex anterior communicating artery aneurysm. (**A**) 3D reconstruction illustrating the sonolucent cranioplasty embedded into the craniotomy directly over the anastomotic site. (**B**) TCUS obtained through the sonolucent window. The image demonstrates patent flow at the anastomotic site (yellow arrow), as well as the callosomarginal vessels (white arrows), interhemispheric fissure (a), and bilateral frontal horns of the lateral ventricles (b). Images are original and obtained from patients at the authors’ institution with appropriate informed consent.

A distinction of clinical importance separates TCUS from conventional bedside Doppler without a sonolucent window. Standard bedside Doppler can detect an audible signal in the proximal STA but cannot differentiate between flow in the bypass limb and flow in non-anastomosed STA branches that may remain intact. A patent, non-bypassed STA branch will produce a reassuring Doppler signal even in the setting of complete bypass occlusion, potentially generating a dangerous false-positive assessment of graft patency [17,58]. TCUS, by directly imaging the anastomosis and the surrounding intracranial vasculature, resolves this issue (Figure 3).

### 5.3. Multicenter Evaluation

The first systematic multicenter validation of TCUS for EC-IC bypass monitoring was provided by Salem et al. in 2023 [18]. Aggregating data from five United States centers over a three-year period (2019–2022), this study enrolled 44 patients undergoing direct EC-IC bypass (the largest published cohort at that time) and was the first to establish generalizability of the technique across different institutions, surgeons, and patient populations.

Qualitative TCUS assessment of graft patency was technically feasible in 100% of cases, a finding that stands in favorable contrast to conventional TCD through native bone, which fails in 8–20% of patients due to inadequate acoustic windows depending on age, sex, and ethnicity. Intraoperative bypass confirmation employed multimodal assessment: microvascular Doppler in 90.9%, indocyanine green (ICG) videoangiography in 86.4%, and catheter angiography in 61.4% of cases, reflecting the real-world practice of complementary rather than exclusive reliance on any single modality [18].

Postoperatively, 56.8% of patients received inpatient TCUS confirmation and 47.7% received outpatient surveillance, proportions representing early institutional adoption rather than an upper limit on feasible utilization. The absence of bypass failures in the cohort produced excellent clinical outcomes but also meant the study could not assess TCUS’ ability to detect a failing graft, the most clinically critical question for any surveillance modality.

The safety profile of the implant was reassuring: the overall complication rate was low, there were no implant-related adverse events, and the single surgical site infection was considered unrelated to the prosthetic material [18]. This safety profile is comparable to or better than autologous bone flap replacement, addressing the theoretical concern that a synthetic implant would increase infection risk.

### 5.4. Longitudinal Performance and Correlation with Angiography

It should be emphasized that, in contrast to the comparatively well-established extracranial duplex and TCD literature, direct evidence for TCUS remains limited to a small number of single-center series, retrospective cohorts, and early multicenter experience, none of which is prospective or randomized. However, the most substantive evidence to date for long-term TCUS reliability and its concordance with formal angiography was published in 2025. This retrospective cohort study included 46 consecutive direct STA-MCA anastomoses in 40 patients at a single high-volume center (March 2021–May 2024) [16]. All surgeries employed a PMMA sonolucent cranioplasty. This study addressed the specific gap left by prior work: the demonstration of TCUS performance not just in the immediate postoperative period, but longitudinally over years of follow-up, and with systematic comparison against formal angiography.

The study population was predominantly female (57.5%), with a mean age of 52.0 years; 55% underwent bypass for ischemic moyamoya disease and 35% for occlusive cerebrovascular disease. There were no cases of postoperative infection, and median clinical follow-up per anastomosis was 1.6 years.

Of the 40 patients, 32 (80%) underwent outpatient TCUS; the lower proportion in the overall cohort reflected evolving institutional practice and inconsistent documentation early in the study period rather than systematic exclusion. The first TCUS was performed at a median of 28.5 days (range 5–117 days), demonstrating patent bypass with robust flow in all 32 cases. A second TCUS was performed in 19 patients (41.3%) at a median of 8.4 months, with patency confirmed in all cases and robust flow demonstrated in 94.7%. Smaller subsets underwent third assessments (n = 5, median 1.2 years) and a fourth assessment (n = 1, 1.4 years), with patency and generally robust flow in all cases. The single case without robust flow at third assessment was the same patient who had demonstrated concerning findings at second assessment, described below.

Follow-up angiography was obtained in 41 of 46 anastomoses (89.1%) at a median of 1.1 years, demonstrating patency in 40 of 41 (97.6%). Among the 31 patients who underwent both TCUS and formal angiography, concordance was 100%, with no false-positive assessments of patency by TCUS. The single bypass occlusion in the cohort, which was identified by DSA after an ischemic stroke 42 days postoperatively, occurred before the patient’s first scheduled TCUS examination, and no TCUS was performed in this case [16]. Thus, similar to the Salem et al. study [18], the absence of bypass failures in the cohort again precluded the chance to test TCUS’ ability to detect a failing graft and remains a major gap in the literature.

Two cases illustrated TCUS’ capacity for hemodynamic characterization beyond binary patency assessment. In one, a second TCUS at 377 days demonstrated a qualitatively attenuated waveform with diminished color Doppler signal; DSA performed the same day confirmed a significantly narrowed anastomosis, and the patient remained asymptomatic on serial follow-up. In the other, a second TCUS identified a markedly reduced STA caliber, subsequently confirmed angiographically. Both cases demonstrate TCUS’ ability to detect evolving hemodynamic changes that correspond to angiographic findings and to prompt timely formal investigation, with both patients remaining clinically stable throughout.

### 5.5. Comparison with Conventional Imaging Modalities

TCUS offers several potential advantages relative to established bypass surveillance modalities. DSA provides unmatched spatial resolution and remains the reference standard for detailed anatomical characterization of anastomotic caliber, suture line integrity, and graft maturation. However, it is invasive, requires arterial catheterization, involves ionizing radiation and iodinated contrast, and carries procedural risks including stroke, arterial injury, and allergic reactions, attributes that limit its suitability for the serial surveillance that bypass patients require, often over many years or decades (Table 1) [16].

CTA offers a less invasive alternative but still involves radiation and contrast, restricting repeated use, particularly in younger patients and those with renal disease [9,48]. MRA avoids radiation, and advanced techniques such as arterial spin labeling-based vessel-selective 4D MRA have shown strong sensitivity and specificity for bypass visualization; however, MRA involves lengthy acquisition times, requires patient transport, and may have difficulty resolving smaller-caliber anastomoses or distinguishing subtle degrees of stenosis with the resolution of DSA [11].

TCUS is noninvasive, radiation-free, requires no contrast, can be performed at the bedside or in the outpatient clinic, and can be repeated without cumulative risk or cost escalation [16,58]. The elimination of acoustic window dependency means that TCUS is technically feasible in virtually all patients who have received a sonolucent implant [64,65,66]. The direct visualization of the anastomotic site provides anatomically specific information unavailable from extracranial duplex or TCD alone [16]. The economic argument for TCUS appears favorable, particularly for programs with high bypass volumes requiring long-term serial follow-up; however, this rests on estimated imaging-cost comparisons rather than formal cost-effectiveness analysis, which has not yet been performed [60].

The multicenter consistency of TCUS performance, showing 100% technical success across five centers, suggests the technique is robust and broadly generalizable rather than dependent on unique expertise at a specialized center [18]. This is an important characteristic for any modality being considered for wider adoption. While prospective data are awaited, the available evidence supports a pragmatic, complementary role for TCUS within existing surveillance pathways. Table 2 outlines clinical scenarios in which TCUS may reasonably serve as a first-line or interval surveillance tool, alongside those in which formal angiographic imaging remains necessary.

### 5.6. Limitations

An honest assessment of TCUS must acknowledge its current limitations. The most fundamental is spatial resolution: TCUS cannot provide the submillimeter anatomical detail of DSA, and it cannot characterize anastomotic caliber, suture line integrity, or the precise degree of stenosis at the level of resolution achievable with catheter angiography [17,58]. The two clinically important cases in the 2025 single-center series in which hemodynamic changes were detected qualitatively by TCUS both ultimately required DSA for definitive anatomical characterization [16]. Thus, TCUS identified the problem but could not fully define it.

Further, the technique is operator-dependent, requiring training in both acquisition technique and image interpretation [16]. Formal interobserver reliability data, standardized training curricula, and objective competency assessment metrics do not yet exist in the published literature. Current TCUS practice is predominantly qualitative, providing near-binary patency assessment supplemented by subjective waveform interpretation. The theoretical potential for quantitative TCUS flow measurement, including vessel diameter, peak systolic velocity, end-diastolic velocity, pulsatility index, and volumetric flow, has not yet been systematically realized in clinical practice, limiting TCUS to a more restricted diagnostic role than is in principle achievable [64].

The additional cost of the PMMA implant relative to autologous bone flap replacement is a barrier to universal adoption. While a comprehensive cost analysis incorporating long-term surveillance imaging costs over the patient’s lifetime might favor the sonolucent implant given the repeated imaging avoided, this analysis has not yet been formally validated [58,60]. The implant is available only in standardized sizes, and anatomical constraints in some patients may limit optimal placement. For high-flow bypasses with long interposition graft courses, TCUS through a single acoustic window can assess only the intracranial anastomotic site, not the full length of the conduit. However, the more proximal segments can be assessed by ultrasound within the neck and deep to soft tissue.

Additionally, the published TCUS evidence base is largely drawn from moyamoya disease and occlusive cerebrovascular disease populations; systematic evaluation in the aneurysm bypass population, where hemodynamic profiles differ, has not yet been specifically reported [16,18,58]. The total body of TCUS-specific literature remains small and is composed predominantly of retrospective single-center series and early multicenter experience, without prospective or comparative validation. Critically, the existing TCUS cohorts reported few or no graft failures; consequently, the sensitivity and specificity of TCUS for detecting true perioperative graft failure, arguably the most clinically important performance characteristic of any surveillance tool, remain essentially unestablished. Reported diagnostic metrics for sonographic bypass assessment (Table 3) overwhelmingly describe prediction of patency or grading of bypass function rather than detection of failure, a distinction that should temper interpretation of the apparent concordance between TCUS and angiography. Lastly, as a narrative rather than systematic review, this article is also subject to selection bias in the studies cited and did not employ a formal risk-of-bias appraisal.

## 6. Future Directions

### 6.1. Quantitative Assessment and Protocol Standardization

The transition from qualitative to quantitative TCUS assessment represents the most important next step. Volumetric flow measurement, anastomotic diameter quantification, and standardized velocity metrics, comparable to those established for extracranial STA duplex, would transform TCUS from a patency surveillance tool into a comprehensive hemodynamic monitoring instrument. Correlation of quantitative Doppler-derived metrics with intraoperative flow data and long-term angiographic findings is an immediate research priority. If successful, TCUS quantitative thresholds analogous to those established for high-flow (>200 mL/min for hyperperfusion risk) and low-flow (Matsushima-grade discriminating thresholds) bypass monitoring by extracranial duplex could be validated for intracranial direct measurement.

Standardization of imaging acquisition protocols is a complementary requirement. Variability in transducer selection, frequency, Doppler angle correction technique, and measurement conventions limits inter-institutional comparability and prevents the establishment of universal reference ranges. Formal training programs and competency standards, comparable to those that exist for diagnostic carotid duplex ultrasonography, are needed as TCUS transitions from early adoption to routine practice. Three-dimensional reconstruction of sonographic images would additionally improve spatial assessment.

### 6.2. Artificial Intelligence Integration

Preliminary work has explored AI-assisted intracranial sonographic image analysis, with early studies demonstrating good concordance between clinician and algorithm-based image quality assessment (intraclass correlation coefficients of 0.829–0.839) [67,68]. The potential applications are broad: automated assessment of image acquisition adequacy, AI-assisted anatomical landmark identification, automated extraction of quantitative flow parameters, and longitudinal comparison of serial examinations to detect subtle interval changes [69]. These capabilities would directly address operator dependency, the most important current limitation of TCUS, and could expand access to high-quality bypass surveillance beyond centers with specialized expertise.

## 7. Conclusions

Sonographic imaging of EC-IC bypass has evolved over four decades from indirect extracranial Doppler waveform assessment to TCUS. Each advance has expanded the temporal and anatomical reach of ultrasound monitoring and reduced dependence on invasive, radiation-exposing angiographic surveillance.

TCUS represents the most significant contemporary advance in this domain. By creating a permanent, surgically implanted acoustic window at the site of the anastomosis, it enables direct real-time visualization of bypass patency and hemodynamics throughout the postoperative course, from the immediate perioperative period through years of long-term follow-up, without radiation, contrast, or patient transport. The multicenter Salem et al. (2023) series demonstrated technical feasibility across institutions, and the longitudinal Grin et al. (2025) cohort established complete concordance with formal angiography over a median follow-up exceeding one year [16,18].

These data support TCUS as a reliable, cost-effective complement to formal angiography for serial bypass surveillance, with formal DSA reserved for cases requiring anatomical detail beyond TCUS resolution or for investigation of concerning ultrasound findings. The high hemodynamic stakes that characterize aneurysm bypass, where the graft may represent the sole source of perfusion for a dependent territory, amplify the clinical value of a reliable, repeatable, noninvasive monitoring tool.

However, key limitations remain. Current assessment is predominantly qualitative, the technique is operator-dependent, and systematic evidence from high-flow and aneurysm-specific bypass populations is limited. These limitations define the next steps in sonographic research: development and validation of standardized quantitative protocols, assessment of interobserver reliability and learning curves, AI-assisted image analysis, and prospective evaluation in the full spectrum of EC-IC bypass indications. As this evidence matures, TCUS appears well-positioned to assume a larger role in routine bypass surveillance, pending prospective validation, with angiographic imaging remaining the reference standard and the appropriate investigation for anatomically complex, hemodynamically equivocal, or symptomatic cases.

## Figures and Tables

**Table 1:** Comparison of imaging modalities for EC-IC bypass surveillance.

Modality	Invasive	Ionizing Radiation	Contrast Required	Bedside/Outpatient Feasible	Spatial Resolution	Direct Anastomosis Visualization	Relative Cost
Digital subtraction angiography (DSA)	Yes (arterial catheterization)	Yes	Yes (iodinated)	No	Highest (reference standard)	Yes	High
Computed tomography angiography (CTA)	Minimal (IV access)	Yes	Yes (iodinated)	No	High	Yes (limited for small anastomoses)	Moderate
Magnetic resonance angiography (MRA)	No	No	Sometimes (gadolinium)	No	Moderate–high; lower for small vessels	Variable; limited for small anastomoses	Moderate–high
Extracranial duplex/transcranial Doppler (TCD)	No	No	No	Yes	Low–moderate; no direct anastomosis view	No (indirect; downstream or donor-vessel only)	Low
Trans-sonolucent cranioplasty ultrasonography (TCUS)	No	No	No	Yes	Lower than DSA; adequate for flow/patency	Yes (through implant window)	Low (estimated)

Footnote: Cost characterizations are qualitative. The only published estimate compares anticipated follow-up imaging cost of approximately US $900 with TCUS versus approximately US $11,000 with conventional modalities (Flores et al. [58]); formal cost-effectiveness analysis has not been performed. TCD/extracranial duplex feasibility is limited by an inadequate temporal acoustic window in approximately 8–20% of patients, whereas TCUS is performed through the sonolucent implant.

**Table 2:** Proposed complementary roles of TCUS and formal angiographic imaging in EC-IC bypass surveillance.

Clinical Scenario	TCUS May Be Appropriate (First-Line/Interval Surveillance)	Formal Angiography (DSA/CTA/MRA) Recommended
Routine asymptomatic longitudinal surveillance with prior reassuring imaging	Yes	Not routinely required if TCUS robust and unchanged
Stable, robust flow on serial TCUS	Yes	Not routinely required
Equivocal, attenuated, or changed TCUS findings	Triage/prompt further imaging	Yes (definitive characterization)
Suspected anastomotic or graft stenosis	Detection/screening	Yes (DSA preferred for caliber and degree of stenosis)
New or recurrent neurological symptoms	Initial bedside assessment	Yes (urgent, to exclude graft failure)
Immediate postoperative period (<24 h)	No (air/wound limits acquisition)	Per institutional protocol
High-flow (interposition graft) bypass	Limited to assessable segments	Yes (full conduit assessment)
Aneurysm bypass with parent-vessel sacrifice	Adjunctive only	Yes (higher stakes; confirm patency and aneurysm exclusion)
Pre-treatment or surgical planning	No	Yes

Footnote: This algorithm reflects the authors’ synthesis of current evidence and is intended as pragmatic guidance pending prospective validation; it is not a validated decision rule.

**Table 3:** Reported diagnostic performance of sonographic parameters for predicting EC-IC bypass patency or grading bypass function.

Study	Technique/Parameter	Threshold	Sensitivity	Specificity	Reported Endpoint
Chen et al. (2023) [12]	Donor STA postoperative PSV	>49 cm/s	92%	83%	Bypass patency (AUC 0.886)
Chen et al. (2023) [12]	Normalized PSV (operated/contralateral)	>1.082	100%	61%	Bypass patency (AUC 0.848)
Chen et al. (2023) [12]	Unadjusted PSV ratio (postop/preop)	>1.218	92%	83%	Bypass patency (AUC 0.899)
Chen et al. (2023) [12]	Adjusted PSV ratio	>1.202	100%	70%	Bypass patency (AUC 0.880)
Kim et al. (2021) [48]	Mean flow-rate ratio (postop/preop) at 1 month	>1.475	95%	100%	Good patency on DSA
Kim et al. (2021) [48]	Cross-sectional diameter ratio at 1 month	>1.15	91%	100%	Good patency on DSA
Nakamizo et al. (2009) [49]	STA diameter at 1 year	≥1.8 mm	75.7%	87.5%	Improved cerebrovascular reserve (>10%); PPV 96.6%, NPV 43.8%
Wang et al. (2020) [13]	Quantitative STA graft flow	124.5 and 65.5 mL/min (Matsushima A/B and B/C)	Not reported	Not reported	Bypass function grade; κ = 0.78 agreement with DSA
Salem et al. (2023) [18]	Qualitative TCUS patency	—	Not assessable	Not assessable	Technical feasibility 100%; no graft failures in cohort
Grin et al. (2025) [16]	Qualitative TCUS patency	—	Not assessable	Not assessable	100% concordance with DSA; no false positives; no graft failures in cohort

Footnote: The reported metrics overwhelmingly describe prediction of patency or grading of bypass function, not detection of true graft failure. Because the TCUS-specific cohorts (Salem et al. [18]; Grin et al. [16]) contained few or no graft failures, the sensitivity and specificity of TCUS for detecting perioperative graft failure cannot be derived from existing data and remain unestablished. For Chen et al. [12], specificity is calculated as 1—reported false-positive rate.

## Data Availability

Due to this manuscript’s nature as a review article, no new data were created.

## Abbreviations

ACA, anterior cerebral artery; CTA, computed tomography angiography; DSA, digital subtraction angiography; EC-IC, extracranial–intracranial; ICA, internal carotid artery; MCA, middle cerebral artery; MRA, magnetic resonance angiography; PCA, posterior cerebral artery; PICA, posterior inferior cerebellar artery; PMMA, polymethyl methacrylate; PSV, peak systolic velocity; SCA, superior cerebellar artery; STA, superficial temporal artery; TCD, transcranial Doppler; TCUS, trans-sonolucent cranioplasty ultrasonography.
